# Fetal Alcohol Exposure: The Common Toll

**DOI:** 10.4172/2329-6488.1000257

**Published:** 2017-02-28

**Authors:** Marie R Nakhoul, Karl E Seif, Natasha Haddad, Georges E Haddad

**Affiliations:** Department of Physiology and Biophysics, College of Medicine, Howard University, Washington, D.C, USA

**Keywords:** Fetal alcohol exposure, Apoptosis, Cardiac defect, Pregnancy, Development, Alcohol and heart, Alcohol and brain, Reactive oxygen species

## Abstract

Alcohol has always been present in human life, and currently it is estimated that 50% of women of childbearing age consume alcohol. It has become increasingly clear over the last years that alcohol exposure during fetal development can have detrimental effects on various organ systems, and these effects are exerted by alcohol through multiple means, including effects on free radical formation, cellular apoptosis, as well as gene expression. Fetal alcohol exposure can lead to a spectrum of short term as well as long-term problems, with Fetal Alcohol Syndrome being on the more severe end of that spectrum. This syndrome is morbid, yet preventable, and is characterized by midfacial hypoplasia, thin upper lip, widely spaced small eyes, long smooth philtrum and inner epicanthal folds. Other findings include growth restriction as well as various neurodevelopmental abnormalities. This article is the first comprehensive review combining the molecular as well as the gross physiological and anatomical effects of alcohol exposure during pregnancy on various organ systems in the body. Our knowledge of these various mechanisms is crucial for our understanding of how alcohol exposure during fetal development can lead to its detrimental effects.

## Introduction

For thousands of years, alcohol has played a major part of human life. Since ancient times, various civilizations have used alcohol for leisure, trade, and religious rites. With the passage of time, multiple civilizations dwindled and vanished, whereas the use of alcohol only grew. In the present time, it is believed that as much as 40% of the world’s adult population consumes alcohol, with an average yearly alcohol consumption of 17.1 L per drinker [1]. Furthermore, it appears that the number of women, in particular, who consume alcohol, has been increasing, whereby it is estimated that 50% of women of child bearing age in the US consume alcohol in various degrees [2]. For non-pregnant women, physicians and many researchers define light drinking as 1.2 drinks per day, moderate drinking as 2.2 drinks per day and heavy drinking as 3.5 or more drinks per day [2]. Moreover, 40% of the 4 million annual pregnancies in the US drink alcohol, and among them 3–5% drink heavily [3]. It wasn’t until 1968 and 1973 that the first descriptions of a fetal syndrome linked to maternal alcohol abuse during pregnancy appeared in modern medical literature [4]. The current medical term for this syndrome is Fetal Alcohol Syndrome (FAS). It includes findings such as midfacial hypoplasia, thin upper lip, widely spaced small eyes, long smooth philtrum and inner epicanthal folds. Other findings include growth restriction as well as various neurodevelopmental abnormalities [2]. The incidence of FAS is estimated to vary between 0.5 and 3/1000 live births in the USA and Canada [5]. Multiple factors are involved in determining the severity of FAS in an afflicted individual. These include: quantity of alcohol consumed during pregnancy, frequency of alcohol use, timing of maternal drinking in terms of gestational age, as well as multiple other maternal risk factors such as age, gravidity, nutrition, socioeconomic status, and metabolic and genetic influence [6,7]. Recent studies have also made clear that the pattern of drinking can also affect the severity of FAS, whereby binge drinking, defined as consuming >48 g of alcohol in one occasion [7], was found to produce very high maternal blood alcohol concentrations, thereby causing the most damage [6]. Given the detrimental effects of alcohol on fetal development, the American Academy of Pediatrics, as well as the American College of Obstetricians and Gynecologists both recommends abstinence during pregnancy for pre-conceptional and pregnant women [2].

Although chronic low alcohol levels have been shown to play a positive effect on cardiac structure and contractility in non-pregnant individuals [8]. This review article aims at covering the varying negative effects of alcohol in pregnancy, specifically on fetal development, based on timing of exposure during pregnancy, as well as the molecular and pathophysiological characteristics of Fetal Alcohol Exposure (FAE) in vital body organs such as the brain, heart and liver. Also, this article will shed light on the long term effects of FAE in the adult life of affected patients. Finally, this review article derives its importance from being the first article to combine the molecular as well as the gross physiological and anatomical effects of alcohol exposure during pregnancy on various organ systems in the body.

## Methods

An extensive web search on PubMed was conducted for articles pertaining to the topic. The following are some of the search terms that were used: Fetal alcohol syndrome, fetal alcohol exposure, apoptosis, alcohol and heart, pregnancy, embryo development, reactive oxygen species. With few exceptions, all the articles were published after the year 2005. 150 articles were carefully read, and from those, information was cited from 73 articles.

## Mechanisms

It is known that the survival of the foetus during pregnancy depends on the exchange of nutrients and respiratory gases with the mother through a very vital gestational organ, the placenta. Along with many other functions, this organ is also known to acts as a barrier to multiple toxins and chemicals, hindering their transmission from maternal blood to fetal blood. However, ethanol can readily cross the placenta, subsequently making its way to fetal blood [9]. Furthermore, alcohol exposure time tends to be prolonged in the fetus due to its decreased capacity to eliminate Ethanol (EtOH) secondary to low fetal alcohol dehydrogenase activity [10] and due to the reabsorption of EtOH that remains trapped in the amniotic fluid [9]. Studies have shown that even moderate amount of alcohol consumption can lead to FAS [9], partly due to this amplification system. Many studies in the literature have attempted to understand the mechanisms by which EtOH can affect fetal development. These mechanisms can be divided into 4 major categories: Molecular, genetic/epigenetic, metabolic, and cellular.

Ethanol (EtOH) has been found to cause apoptosis of neural crest cells [11], a group of progenitor cells that can give rise to a multitude of cell types including neurons, glial cells, and mesenchymal cells [12]. This ethanol-induced apoptosis has been shown to be mediated through different molecular mechanisms, one of which is increased mRNA expression of the Siah1 protein, a member of the E3 ubiquitin ligase family [11], which triggers apoptosis through phosphorylation of p38 MAPK, leading to activation of the p53 signaling pathway. p53 is known to be a transcription factor for many pro-apoptotic genes [13]. Furthermore, ethanol has been found to be involved in other apoptotic signaling pathways, including Fas/Fas-L [14], Bcl-2 family [15], and caspase signaling systems [16]. However, these mechanisms have not been clearly elucidated [13].

**Figure 1:**
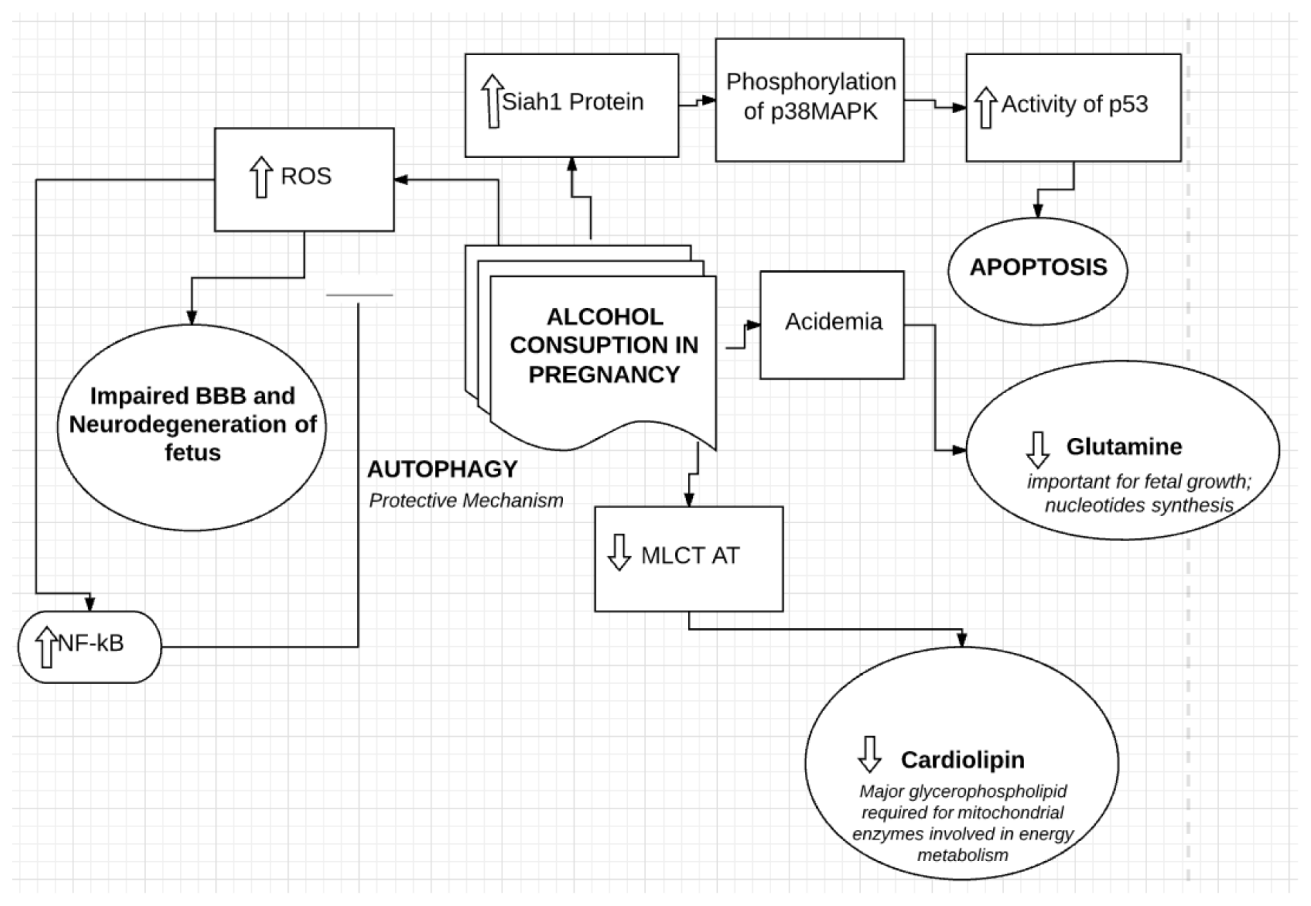
Effect of alcohol exposure on fetus. MLCT AT: Monolysocardiolipin acyltransferase; BBB: Blood Brain Barrier; ROS: Reactive Species; Legend⬇: Decreases;⬆: Increases; →, leads to ⊣: inhibits. Explanation: Alcohol consumption in pregnancy leads to: 1) Decrease in MLCT AT which will lead to decrease in cardiolipin ( a major glycerophospholipid required for mitochondrial enzymes involved in energy metabolism; 2) Acedemia which will decrease glutamine which is important for fetal growth and nucleotides synthesis; 3) Increase in Siah1 protein leading to phosphorylation of p38MAPK leading to increase activity of p 53 and eventually apoptosis; 4) Increase in ROS which leads to an: a) impairment of BBB leading to Neurodegeneration of fetus; b) increase in NF-kB which will inhibit the increase of ROS through a protective mechanism (autophagy).

In addition to its role in apoptosis, EtOH has been found to affect cellular respiration in rat cardiac cells by influencing the mitochondrial membrane, specifically Cardiolipin, a major glycerol-phospholipid required for mitochondrial enzymes involved in energy metabolism within the cell [17]. This latter mechanism has been explained by the fact that metabolites of alcohol, specifically fatty acid ethyl esters, decrease the activity of monolysocardiolipin acyltransferase (MLCL AT), which is the rate-limiting enzyme in the transformation of monolysocardiolipin to Cardiolipin in cardiac mitochondria [17]. In fact, Taylor et al., found that the activity of the MLCT AT enzyme was 36% lower in newborn rats with in-utero exposure to alcohol [10]. Also, not only do fatty acid ethyl esters decrease the activity of MLCL AT, but they also seem to play a role in altering protein expression and uncoupling oxidative phosphorylation by directly binding to mitochondria in intact cells [18]. Furthermore, a third mechanism is alcohol mediated oxidative damage. An example of this is the increased production of Reactive Oxygen Species (ROS) in the brain by catalase mediated acetaldehyde production from alcohol, thus leading to impairment of the blood brain barrier and neurodegeneration in the developing fetus [19–21]. A fourth major mechanism by which EtOH has been found to affect fetal development, apart from apoptosis, oxidative stress, and cellular respiration, is acidemia mediated decrease in key amino acid concentrations, especially that of Glutamine which plays a role of paramount importance in fetal growth, as well as synthesis of nucleotides [22], the antioxidant glutathione [23], and the brain neurotransmitter glutamate [23]. This alcohol mediated reduction in glutamine and other related amino acids in the plasma were found to be around 21–30% in exposed pregnant Ewes as per a study done by Washburn et al. [24]. It has been found that blood pH decreases proportionally to increases in ethanol blood concentration since ethanol consumption leads to a mixed respiratory and metabolic acidosis [25]. This acidosis leads to increased glutamine uptake and metabolism by renal mitochondria [26]. Acidosis also increases renal expression of N-glutamine transporter (SNAT 3), which increases glutamine uptake by the kidney [27]. When it comes to binge drinking, Ramadoss et al. [28] created a Ewe model mimicking third trimester binge drinking, and showed acidosis mediated drop in plasma glutamine and other amino acid levels including threonine, serine, glutamine, glycine, alanine and methionine [29]. This drop in amino acids directly affects the synthesis of various functional and structural proteins (Figure 1).

Concerning the genetic aspect, it is known that the relationship between alcohol and genetics goes both ways: On one hand, an individual’s genetic makeup can have a direct effect on alcohol metabolism and hence alcohol blood concentration level. Various enzymes are involved in the metabolism of alcohol, such as alcohol dehydrogenase, aldehyde dehydrogenase, and CYP2E1 [30]. Different variations of the genes encoding for these enzymes in the mother and fetus can lead to differences in enzyme activity and hence differences in alcohol metabolism and alcohol related damage [30]. On the other hand, alcohol has been shown to affect gene expression without having direct effects on the gene sequence itself [31]. This is referred to as epigenetic processes, including DNA methylation, histone modification and RNA interactions. Starting with DNA methylation, it is the process of transferring a methyl group by DNA methyltransferase to one of the four DNA nucleotides, thereby leading to a condensed chromatin formation, thus effectively silencing gene expression [32]. It is folate and B12, and their involvement in the formation of SAM (s-adenosyl methionine), that are involved in the supply of these methyl groups [33]. Alcohol excess is known to affect folate metabolism and availability, subsequently influencing DNA methylation, affecting fetal growth and development [34]. Furthermore, alcohol’s effect on folate levels also impacts histone activity, as folate is known to be involved in histone methylation [34]. Histones are principle structural proteins of chromosomes in eukaryotes, and certain histone types are involved in the formation of DNA-histone units called nucleosomes [34]. The interaction of these nucleosomes with other histone types is what determines the transcriptional activity of chromatin. Alcohol has been shown to lead to hypermethylation of Lysine 4 in histone 3 and hypomethylation of Lysine 4 in histone 9 in studies done on hepatocytes [34]. It is suspected that these distinct methylation changes in histones can affect chromatin structure, and thus gene expression level [34]. Other alcohol related changes in histone include histone acetylation, a process that has epigenetic effects on various organs. It was demonstrated that histone acetylation in lung cells leads to increased apoptosis [35]. In the amygdala, histone acetylation was shown to change gene expression of Neuropeptide Y, an important molecule in the autonomic nervous system [36]. Also, in the heart, an experiment done by Pan et al. demonstrated that H3 hyperacetylation could be linked to structural heart defects such as biventricular enlargement and septal thinning [37,38]. Zong et al. (2010) similarly found that both low and high levels of alcohol lead to hyperacetylation of histones; but interestingly, it is only at high levels that genes involved in cardiac structure such as GATA 4, Mef2C, Tbx5 are affected [38].

Given the various mechanisms by which alcohol induces damage to cellular development and function, the body has devised various protective mechanisms to hinder the damaging effects of alcohol. One such mechanism is neuronal cell autophagy, whereby ethanol-induced ROS (mainly originating from NADPH oxidase) regulate the activities of certain transcriptional factors such as NFKB, TP53 and NFE2L2. These factors affect various signaling pathways involved in autophagy and apoptosis of neuronal cells, thereby blocking ROS induced neurodegeneration [39]. Chen et al. touched upon one of the autophagy signaling pathways, reporting that ethanol enhances autophagy through inhibition of the mTOR (Mammalian Target of Rapamycin) pathway [40]. Furthermore, it was found that ethanol leads to a moderate increase in the expression of Nrf2, a main regulator of expression of antioxidant genes. It is important to note that it has been shown that this moderate increase in Nrf2 in relation to ethanol is only an adaptive response, and that this response is not enough to prevent ethanol induced apoptosis [41]. However, understanding these protective mechanisms against alcohol is vital, since it sheds light on possible treatments that can make use of these mechanisms to hinder the negative effects of alcohol.

Finally, not only does ethanol damage cells by various mechanisms, but it is suspected to also have varying effects, depending on the gestational age when ethanol exposure occurs. This has been studied in literature at the level of neuronal cells. It has been shown that certain regions such as the ventromedial forebrain and mesodermal cells are affected when EtOH exposure occurs in the first trimester. On the other hand, third trimester exposure to EtOH seems to affect different brain regions such as the prefrontal cortex, hippocampus, cerebellum as well as the corpus callosum [42,43].

## Pathophysiology

After reviewing the various major mechanisms by which EtOH mediates its detrimental effects on fetal development, we will now proceed with the discussion of the various physiological and anatomical effects of alcohol on different organ systems, specifically on organs affected most by alcohol exposure such as the liver, the heart, and the brain.

Beginning with the liver, earlier studies have shown that children with fetal alcohol syndrome may have abnormal liver function tests, along with fibrosis and fatty hepatic degeneration [44]. Furthermore, it was demonstrated increased fat deposits in hepatic cells of rat fetuses when they were exposed to alcohol in late trimesters [45]. However, more recent studies on sheep by Sozo et al. have failed to show any change in fetal liver morphology [46]. The effect of alcohol in these studies was found to affect iron homeostasis with change in gene expression of hepcidin and ferroportin, and subsequent drop in fetal liver ferric content when alcohol exposure occurred in the third trimester [46]. A prospective study by Carter et al. also demonstrated the effect of alcohol on iron homeostasis, whereby there was a 3.6 times increased risk of iron deficiency anemia in infants of mothers who binge drank during pregnancy. This was explained by possible disruption of iron transport to the fetus via the placenta, in addition to the disruption of the absorption and storage of iron by the fetus. Interestingly, it was observed that alcohol exposed infants with iron deficiency anemia had delayed growth, and had a lag in weight gain and head circumference during the first year of life, and these delays could not be corrected after replacing iron stores in the newborn, suggesting that iron could be one of the instigating factors. However, sustainable effects of alcohol on infant growth seem to be driven to a greater extent through mechanisms other than iron deficiency [47].

In the brain, alcohol has been found to have both structural and functional effects on neural development. These include decreased brain volumes, defects in development of certain brain structures like the brainstem, cerebellum, corpus callosum, frontal lobe, and thalamic nuclei, as well as errors in the migration of neurons [48,49]. Radial glial cells, which are precursors for various cells within the nervous system such as neurons, astrocytes, and oligodendrocytes, are disrupted when exposed to alcohol [50]. Prenatal alcohol exposure has also been shown to reduce connectivity between the two hemispheres of the brain, notably in the para-central cortical regions, thereby delaying the maturation of brain networks, while simultaneously increasing the connectivity between somatosensory, motor, and brainstem networks [51,52]. Burke et al. demonstrated that alcohol exposure in monkeys during the third trimester led to a decrease in the number of neural progenitor cells in the olfactory bulb and dentate gyrus [53]. Cortical development has also been shown to be affected by alcohol exposure during fetal development by altering the migration of Gamma-Aminobutyric Acid (GABA) secreting interneurons that mediate the inhibitory/excitatory balance within the intracoritcal circuit [54]. Furthermore, children who have been exposed to alcohol in-utero were found to have difficulties in executive functioning, including difficulties in organizing thoughts, maintaining emotional stability, planning, and self-monitoring [49]. Motor impairment has also been described, whereby affected children were noted to have delays in fine motor skills, hand-eye coordination, and motor reaction time to visual stimuli [55,56]. Attention and learning capacity have also been affected by alcohol exposure in utero, and interestingly FAE children have been known to be misdiagnosed with Attention Deficit Hyperactivity Disorder (ADHD) due to their irritable behaviors and difficulty in maintaining attention [57]. Finally, the effects of prenatal alcohol exposure also include problems with social interactions and overall social skills, including hyperactive behaviors, communication difficulties, and problems with assuming responsibility, with more severe social impairment seen in females as compared to males [58].

As for the cardiovascular system, it is important to note that this complex organ has multiple components, each originating from different precursor cells [59]. These components include cardiomyocytes, connective tissues, conduction system cells, as well as smooth muscle cells and endothelial cells [59]. The three sources of cardiac cell precursors are the cardiogenic mesoderm cells, the proepicardium, and the cardiac neural crest cells [59]. The latter specifically gives rise to the autonomic nervous system of the heart, along with playing a crucial role in heart septation and valvular development [59]. The importance of neural crest cells has been demonstrated in studies that showed a reduced great vessel diameter and atrioventricular valve leaflet volumes in embryos where these precursor cells were ablated. Interestingly, these heart defects were found to be similar to those seen in avian models of fetal alcohol syndrome, thus proposing that one possible mechanism for alcohol induced cardiac damage is through abnormal development of neural crest cells [60]. A possible explanation for the link between alcohol exposure and neural crest cell dysfunction could be that these cells tend to have higher sensitivity to free radicals, and thus higher rates of apoptosis, due to an inherent decreased endogenous superoxide dismutase activity [61–63]. Other studies have also proven that alcohol exposure can hinder neural crest cell migration [63]. Furthermore, studies performed on zebra fish have demonstrated that differences in the length of ethanol exposure can lead to different degrees of cardiac abnormalities during fetal development, whereby chronic alcohol exposure was more likely to lead to significantly more severe endocardial cushion defects as compared to defects seen in short term alcohol exposure. In fact, hearts exposed to alcohol chronically during development were found to be small with no internal tissue separating the chambers, due to the elimination of endocardial cushion cells [64]. Apart from the role of alcohol exposure length, avian models have also demonstrated the role of ethanol concentration in determining the degree of cardiac effects, whereby valvular abnormalities were found in 68% of hearts from embryos exposed to high concentrations of alcohol [65]. Similarly, studies performed on larvae showed that exposure to higher concentrations of alcohol led to drastic dorsal aorta destruction and segmental artery coarctation. Also, hearts of the high alcohol concentration exposure group showed morphological differences and were significantly smaller than the control group [66]. However, even in low alcohol concentrations, the heart rates of the exposed hearts were found to be slower than the control hearts, which is more pronounced in hearts exposed to higher concentrations [67].

After discussing the effects of alcohol exposure on various organ systems during fetal development, it is important to shed light on more lasting effects of in utero alcohol exposure. In fact, multiple studies have demonstrated that there are long-term consequences of fetal alcohol exposure, which has been described by the Barker Hypothesis. Barker suggested that certain patterns of fetal growth could affect various body functions including blood pressure control, sensitivity to insulin, metabolism of glucose, as well as cardiac functions in adult life. Disruptions to these patterns of growth by factors like maternal malnutrition, stress, and alcohol exposure can affect these aforementioned physiological functions on the long term. This hypothesis has also been termed the “Developmental Origins of Adult Health and Disease” or “DOHaD” [68–71]. Furthermore, studies have shown that early life ethanol exposure can affect mesenchymal stem cells, the main cells responsible for tissue repair, thus increasing susceptibility to disease later on in life. Leu et al. found that mesenchymal stem cells of rats exposed to ethanol during the third trimester equivalent were resistant to osteogenic and adipogenic inductions in comparison to controls. In these experiments, it was found that expression of multiple proteins like osteocalcin and alkaline phosphatase was impaired [72]. Other experiments on mouse models have shown consequences like reduced weight, survival, and immune response, specifically B and T cell response, in alcohol-exposed mice. Furthermore, there were an increased number of tumors in those mice as compared to controls. Other observed effects included increased insulin resistance, hypertriglyceridemia, as well as increased chronic inflammatory processes such as arthritis [73]. Given these devastating effects, we believe that it is crucial that fetal alcohol exposure is identified early on by obstetrician and gynecologists as well as pediatricians/neonatologist, in order to actively manage and prevent the long-term sequelae.

## Conclusion

Alcohol use has been steadily increasing, and its consumption by women continues to be on the rise. All evidence shows that alcohol plays a detrimental effect on fetal development. As discussed extensively in this article, these effects span multiple organ systems from the heart and brain to the liver, kidneys, as well as other major organs. Alcohol exerts these effects in multiple ways, with influences on free radical formation, cell programming and cell death, as well as gene expression, among many other mechanisms. The exposed body reacts by certain adaptive responses, such as an increase in Nrf2, which affects the expression of anti-oxidant genes, but this increase is not sufficient to prevent ethanol induced apoptosis. It is also clear that the harmful effects of alcohol vary in relation to the pattern of drinking, with more harm done with binge drinking than with chronic alcohol use, as well as to the level of alcohol exposure during pregnancy. Furthermore, there is a temporal component to alcohol-mediated damage, whereby exposure at a specific gestational age during the pregnancy can lead to a variety in both nature and severity of the effect. The information presented in this article is crucial for our understanding of the development as well as the prevention of the effects of fetal alcohol exposure, which are morbid, yet preventable, and affecting thousands of individuals worldwide, and leading to chronic medical, developmental, and social problems in affected patients. Effort needs to be done in order to increase awareness about the harmful effects of even minimal amounts of alcohol consumption during pregnancy. Women need to be counselled to stop alcohol intake completely during their preconception visits, and the question of whether or not they are consuming alcohol while pregnant needs to be brought up during every prenatal follow up visit with the obstetrician. Furthermore, more research is needed to uncover hidden pathways through which alcohol can affect cell structure and function. Understanding these different pathways and attempting to block them may eventually allow us to mitigate the harmful effects of alcohol exposure during fetal development. It is important to note that many cases of fetal alcohol exposure are discovered later in life, sometimes even during adulthood. This is due to the epigenetic effects of alcohol, leading sometimes to late manifestations of abnormal development. Therefore, more effort needs to be made in order to discover fetal alcohol exposure as early as possible and subsequently managing the chronic effects early on and attempt to decrease the morbidity. Currently, it is estimated that 40000 children in the U.S. are born with FASD every year. This figure represents 2–5% of school-aged children. This is preventable mainly through education for parents, and through raising awareness of the harmful effects of alcohol during pregnancy. Also, training and guidance could be provided for medical professionals in the field of women’s health to properly council women of childbearing age. Schools also play a role by creating special needs programs so that affected children are capable of reaching their full educational potential [74]. Finally, we conclude by saying that alcohol consumption during pregnancy has a toll, shared by both affected individuals as well as the society in which they live, given the various socioeconomic effects that Fetal Alcohol Exposure has. Therefore, it requires a collective effort in order to avoid paying this toll and to ensure the health of the future generations to come.

## References

[1] Shield KD, Rylett M, Gmel G, Gmel G, Kehoe-Chan TA (2013). Global alcohol exposure estimates by country, territory and region for 2005--a contribution to the Comparative Risk Assessment for the 2010 Global Burden of Disease Study. Addiction.

[2] Sokol RJ, Delaney-Black V, Nordstrom B (2003). Fetal alcohol spectrum disorder. JAMA.

[3] Floyd RL, Sidhu JS (2004). Monitoring prenatal alcohol exposure. Am J Med Genet C Semin Med Genet.

[4] Hoyme HE, May PA, Kalberg WO, Kodituwakku P, Gossage JP (2005). A practical clinical approach to diagnosis of fetal alcohol spectrum disorders: clarification of the 1996 institute of medicine criteria. Pediatrics.

[5] Farag M (2014). Diagnostic issues affecting the epidemiology of fetal alcohol spectrum disorders. J Popul Ther Clin Pharmacol.

[6] May PA, Gossage JP (2011). Maternal risk factors for fetal alcohol spectrum disorders: not as simple as it might seem. Alcohol Res Health.

[7] Yang J, Qiu H, Qu P, Zhang R, Zeng L (2015). Prenatal Alcohol Exposure and Congenital Heart Defects: A Meta-Analysis. PLoS One.

[8] Rodriguez A, Chawla K, Umoh NA, Cousins VM, Ketegou A (2015). Alcohol and Apoptosis: Friends or Foes?. Biomolecules.

[9] Khalid O, Kim JJ, Kim HS, Hoang M, Tu TG (2014). Gene expression signatures affected by alcohol-induced DNA methylomic deregulation in human embryonic stem cells. Stem Cell Res.

[10] Taylor WA, Legare D, Lautt WW, Hatch GM (2007). Regulation of cardiac mitochondrial monolysocardiolipin acyltransferase activity and expression during development and in fetal alcohol syndrome. Proc West Pharmacol Soc.

[11] Sun H, Chen X, Yuan F, Liu J, Zhao Y (2014). Involvement of seven in absentia homolog-1 in ethanol-induced apoptosis in neural crest cells. Neurotoxicol Teratol.

[12] Delfino-Machin M, Chipperfield TR, Rodrigues FS, Kelsh RN (2007). The proliferating field of neural crest stem cells. Dev Dyn.

[13] Yuan F, Chen X, Liu J, Feng W, Wu X (2016). Up-regulation of Siah1 by ethanol triggers apoptosis in neural crest cells through p38 MAPK-mediated activation of p53 signaling pathway. Arch Toxicol.

[14] Jana K, Jana N, De DK, Guha SK (2010). Ethanol induces mouse spermatogenic cell apoptosis in vivo through over-expression of Fas/Fas-L, p53, and caspase-3 along with cytochrome c translocation and glutathione depletion. Mol Reprod Dev.

[15] Hong F, Kim WH, Tian Z, Jaruga B, Ishac E (2002). Elevated interleukin-6 during ethanol consumption acts as a potential endogenous protective cytokine against ethanol-induced apoptosis in the liver: involvement of induction of Bcl-2 and Bcl-x(L) proteins. Oncogene.

[16] Han JY, Joo Y, Kim YS, Lee YK, Kim HJ (2005). Ethanol induces cell death by activating caspase-3 in the rat cerebral cortex. Mol Cells.

[17] Hatch GM (2004). Cell biology of cardiac mitochondrial phospholipids. Biochem Cell Biol.

[18] Lugea A, Gukovsky I, Gukovskaya AS, Pandol SJ (2003). Nonoxidative ethanol metabolites alter extracellular matrix protein content in rat pancreas. Gastroenterology.

[19] Mansouri A, Demeilliers C, Amsellem S, Pessayre D, Fromenty B (2001). Acute ethanol administration oxidatively damages and depletes mitochondrial dna in mouse liver, brain, heart, and skeletal muscles: protective effects of antioxidants. J Pharmacol Exp Ther.

[20] Haorah J, Schall K, Ramirez SH, Persidsky Y (2008). Activation of protein tyrosine kinases and matrix metalloproteinases causes blood-brain barrier injury: Novel mechanism for neurodegeneration associated with alcohol abuse. Glia.

[21] Chater-Diehl EJ, Laufer BI, Castellani CA, Alberry BL, Singh SM (2016). Alteration of Gene Expression, DNA Methylation, and Histone Methylation in Free Radical Scavenging Networks in Adult Mouse Hippocampus following Fetal Alcohol Exposure. PLoS One.

[22] Kwon H, Spencer TE, Bazer FW, Wu G (2003). Developmental changes of amino acids in ovine fetal fluids. Biol Reprod.

[23] Mates JM, Perez-Gomez C, Nunez de Castro I, Asenjo M, Marquez J (2002). Glutamine and its relationship with intracellular redox status, oxidative stress and cell proliferation/death. Int J Biochem Cell Biol.

[24] Washburn SE, Sawant OB, Lunde ER, Wu G, Cudd TA (2013). Acute alcohol exposure, acidemia or glutamine administration impacts amino acid homeostasis in ovine maternal and fetal plasma. Amino Acids.

[25] Ramadoss J, Wu G, Cudd TA (2008). Chronic binge ethanol-mediated acidemia reduces availability of glutamine and related amino acids in maternal plasma of pregnant sheep. Alcohol.

[26] Nissim I (1999). Newer aspects of glutamine/glutamate metabolism: the role of acute pH changes. Am J Physiol.

[27] Moret C, Dave MH, Schulz N, Jiang JX, Verrey F (2007). Regulation of renal amino acid transporters during metabolic acidosis. Am J Physiol Renal Physiol.

[28] Ramadoss J, Hogan HA, Given JC, West JR, Cudd TA (2006). Binge alcohol exposure during all three trimesters alters bone strength and growth in fetal sheep. Alcohol.

[29] Padmanabhan R, Ibrahim A, Bener A (2002). Effect of maternal methionine pre-treatment on alcohol-induced exencephaly and axial skeletal dysmorphogenesis in mouse fetuses. Drug Alcohol Depend.

[30] Gemma S, Vichi S, Testai E (2007). Metabolic and genetic factors contributing to alcohol induced effects and fetal alcohol syndrome. Neurosci Biobehav Rev.

[31] Ungerer M, Knezovich J, Ramsay M (2013). In utero alcohol exposure, epigenetic changes, and their consequences. Alcohol Res.

[32] Rodenhiser D, Mann M (2006). Epigenetics and human disease: translating basic biology into clinical applications. CMAJ.

[33] Friso S, Choi SW, Girelli D, Mason JB, Dolnikowski GG (2002). A common mutation in the 5,10-methylenetetrahydrofolate reductase gene affects genomic DNA methylation through an interaction with folate status. Proc Natl Acad Sci U S A.

[34] Pal-Bhadra M, Bhadra U, Jackson DE, Mamatha L, Park PH (2007). Distinct methylation patterns in histone H3 at Lys-4 and Lys-9 correlate with up- & down-regulation of genes by ethanol in hepatocytes. Life Sci.

[35] Wang X, Gomutputra P, Wolgemuth DJ, Baxi LV (2010). Acute alcohol exposure induces apoptosis and increases histone H3K9/18 acetylation in the mid-gestation mouse lung. Reprod Sci.

[36] Pandey SC, Ugale R, Zhang H, Tang L, Prakash A (2008). Brain chromatin remodeling: a novel mechanism of alcoholism. J Neurosci.

[37] Pan B, Zhu J, Lv T, Sun H, Huang X (2014). Alcohol consumption during gestation causes histone3 lysine9 hyperacetylation and an alternation of expression of heart development-related genes in mice. Alcohol Clin Exp Res.

[38] Zhong L, Zhu J, Lv T, Chen G, Sun H (2010). Ethanol and its metabolites induce histone lysine 9 acetylation and an alteration of the expression of heart development-related genes in cardiac progenitor cells. Cardiovasc Toxicol.

[39] Luo J (2014). Autophagy and ethanol neurotoxicity. Autophagy.

[40] Chen G, Ke Z, Xu M, Liao M, Wang X (2012). Autophagy is a protective response to ethanol neurotoxicity. Autophagy.

[41] Chen X, Liu J, Chen SY (2013). Over-expression of Nrf2 diminishes ethanol-induced oxidative stress and apoptosis in neural crest cells by inducing an antioxidant response. Reprod Toxicol.

[42] Olney JW, Tenkova T, Dikranian K, Qin YQ, Labruyere J (2002). Ethanol-induced apoptotic neurodegeneration in the developing C57BL/6 mouse brain. Brain Res Dev Brain Res.

[43] Sulik KK (2005). Genesis of alcohol-induced craniofacial dysmorphism. Exp Biol Med (Maywood).

[44] Habbick BF, Zaleski WA, Casey R, Murphy F (1979). Liver abnormalities in three patients with fetal alcohol syndrome. Lancet.

[45] Addolorato G, Gasbarrini A, Marcoccia S, Simoncini M, Baccarini P (1997). Prenatal exposure to ethanol in rats: effects on liver energy level and antioxidant status in mothers, fetuses, and newborns. Alcohol.

[46] Sozo F, Dick AM, Bensley JG, Kenna K, Brien JF (2013). Alcohol exposure during late ovine gestation alters fetal liver iron homeostasis without apparent dysmorphology. Am J Physiol Regul Integr Comp Physiol.

[47] Carter RC, Jacobson SW, Molteno CD, Jacobson JL (2007). Fetal alcohol exposure, iron-deficiency anemia, and infant growth. Pediatrics.

[48] Jones KL, Smith DW (1973). Recognition of the fetal alcohol syndrome in early infancy. Lancet.

[49] Fryer SL, Tapert SF, Mattson SN, Paulus MP, Spadoni AD (2007). Prenatal alcohol exposure affects frontal-striatal BOLD response during inhibitory control. Alcohol Clin Exp Res.

[50] Wilhelm CJ, Guizzetti M (2015). Fetal Alcohol Spectrum Disorders: An Overview from the Glia Perspective. Front Integr Neurosci.

[51] Donald KA, Ipser JC, Howells FM, Roos A, Fouche JP (2016). Interhemispheric Functional Brain Connectivity in Neonates with Prenatal Alcohol Exposure: Preliminary Findings. Alcohol Clin Exp Res.

[52] Wozniak JR, Mueller BA, Bell CJ, Muetzel RL, Hoecker HL (2013). Global functional connectivity abnormalities in children with fetal alcohol spectrum disorders. Alcohol Clin Exp Res.

[53] Burke MW, Inyatkin A, Ptito M, Ervin FR, Palmour RM (2016). Prenatal Alcohol Exposure Affects Progenitor Cell Numbers in Olfactory Bulbs and Dentate Gyrus of Vervet Monkeys. Brain Sci.

[54] Skorput AG, Yeh HH (2016). Chronic Gestational Exposure to Ethanol Leads to Enduring Aberrances in Cortical Form and Function in the Medial Prefrontal Cortex. Alcohol Clin Exp Res.

[55] Mattson SN, Crocker N, Nguyen TT (2011). Fetal alcohol spectrum disorders: neuropsychological and behavioral features. Neuropsychol Rev.

[56] Simmons RW, Thomas JD, Levy SS, Riley EP (2006). Motor response selection in children with fetal alcohol spectrum disorders. Neurotoxicol Teratol.

[57] Roszel EL (2015). Central nervous system deficits in fetal alcohol spectrum disorder. Nurse Pract.

[58] Rasmussen C, Becker M, McLennan J, Urichuk L, Andrew G (2011). An evaluation of social skills in children with and without prenatal alcohol exposure. Child Care Health Dev.

[59] Brade T, Pane LS, Moretti A, Chien KR, Laugwitz KL (2013). Embryonic heart progenitors and cardiogenesis. Cold Spring Harb Perspect Med.

[60] Ma P, Gu S, Karunamuni GH, Jenkins MW, Watanabe M (2016). Cardiac neural crest ablation results in early endocardial cushion and hemodynamic flow abnormalities. Am J Physiol Heart Circ Physiol.

[61] Davis WL, Crawford LA, Cooper OJ, Farmer GR, Thomas DL (1990). Ethanol induces the generation of reactive free radicals by neural crest cells in vitro. J Craniofac Genet Dev Biol.

[62] Chen SY, Sulik KK (1996). Free radicals and ethanol-induced cytotoxicity in neural crest cells. Alcohol Clin Exp Res.

[63] Smith SM, Garic A, Flentke GR, Berres ME (2014). Neural crest development in fetal alcohol syndrome. Birth Defects Res C Embryo Today.

[64] Sarmah S, Marrs JA (2013). Complex cardiac defects after ethanol exposure during discrete cardiogenic events in zebrafish: prevention with folic acid. Dev Dyn.

[65] Serrano M, Han M, Brinez P, Linask KK (2010). Fetal alcohol syndrome: cardiac birth defects in mice and prevention with folate. Am J Obstet Gynecol.

[66] Li X, Gao A, Wang Y, Chen M, Peng J (2016). Alcohol exposure leads to unrecoverable cardiovascular defects along with edema and motor function changes in developing zebrafish larvae. Biol Open.

[67] Lunde ER, Washburn SE, Golding MC, Bake S, Miranda RC (2016). Alcohol-Induced Developmental Origins of Adult-Onset Diseases. Alcohol Clin Exp Res.

[68] Barker DJ (2004b). The developmental origins of chronic adult disease. Acta Paediatr Suppl.

[69] Barker DJ (2004a). Developmental origins of adult health and disease. J Epidemiol Community Health.

[70] Barker DJ (2007). The origins of the developmental origins theory. J Intern Med.

[71] Barker DJ (2004c). The developmental origins of well-being. Philos Trans R Soc Lond B Biol Sci.

[72] Leu YW, Chu PY, Chen CM, Yeh KT, Liu YM (2014). Early life ethanol exposure causes long-lasting disturbances in rat mesenchymal stem cells via epigenetic modifications. Biochem Biophys Res Commun.

[73] Moore EM, Riley EP (2015). What Happens When Children with Fetal Alcohol Spectrum Disorders Become Adults?. Curr Dev Disord Rep.

[74] Wilhoit LF, Scott DA, Simecka BA (2017). Fetal Alcohol Spectrum Disorders: Characteristics, Complications, and Treatment. Community Ment Health J.

